# Clustering of conformational IgE epitopes on the major dog allergen Can f 1

**DOI:** 10.1038/s41598-017-11672-5

**Published:** 2017-09-22

**Authors:** Mirela Curin, Milena Weber, Gerhard Hofer, Danijela Apostolovic, Walter Keller, Renate Reininger, Ines Swoboda, Susanne Spitzauer, Margit Focke-Tejkl, Marianne van Hage, Rudolf Valenta

**Affiliations:** 1 Division of Immunopathology, Department of Pathophysiology and Allergy Research, Center for Pathophysiology, Infectiology and Immunology, Medical University of Vienna, Vienna, Austria; 2 Institute of Molecular Biosciences, University of Graz, Graz, Austria; 3 Immunology and Allergy Unit, Department of Medicine Solna, Karolinska Institute and University Hospital, Stockholm, Sweden; 4 Department of Laboratory Medicine, Medical University of Vienna, Vienna, Austria; 5 Present Address: Molecular Biotechnology Section, University of Applied Sciences, Campus Vienna Biocenter, Vienna, Austria

## Abstract

Immunoglobulin E (IgE)-associated allergy affects more than 25% of the population. Can f 1 is the major dog allergen associated with respiratory symptoms but the epitopes recognized by allergic patients IgE on Can f 1 are unknown. To characterize IgE epitopes of Can f 1 recognized by dog allergic patients, six overlapping peptides spanning the Can f 1 sequence were synthesized. In direct IgE epitope mapping experiments peptides were analyzed for IgE reactivity by dot blot and Enzyme-linked immunosorbent assay (ELISA) with sera from dog allergic patients. For indirect epitope-mapping, rabbits were immunized with the peptides to generate specific IgG antibodies which were used to inhibit allergic patients’ IgE binding to Can f 1. IgE binding sites were visualized on a model of the Can f 1 three-dimensional structure. We found that Can f 1 does not contain any relevant sequential IgE epitopes. However, IgE inhibition experiments with anti-peptide specific IgGs showed that Can f 1 N- and C-terminal portion assembled a major conformational binding site. In conclusion, our study is the first to identify the major IgE epitope-containing area of the dog allergen Can f 1. This finding is important for the development of allergen-specific treatment strategies.

## Introduction

IgE-associated allergy is the most common hypersensitivity disease affecting more than 25% of the population worldwide. The symptoms of allergy include hayfever, asthma, skin inflammation, food allergy and life-threatening systemic anaphylactic reactions. Allergy to furry animals, in particular to cat and dog, is a major risk factor for the development of asthma and rhinitis^1,2^. Dogs are kept as household pets worldwide but dog allergens were also found in homes without dogs, in schools and other public places and it is therefore almost impossible to avoid dog contact^3,4^. Dog-induced allergic symptoms range from rhinoconjuctivitis to severe asthma attacks^1,2^. In a large study conducted in a European population of patients with suspected allergic disease 27% showed positive skin prick test reactions with dog extract^5^. In a cross-sectional population based study in Germany up to 9% of children and adolescents showed sensitization to dog dander which is in good agreement with data obtained for the Swedish birth cohort BAMSE^6,7^. Most of the mammalian-derived allergens, including several dog allergens, belong to the lipocalin protein family^2^. Can f 1 is the major dog allergen with an IgE recognition prevalence of 49–76% in dog allergic populations^8–10^. Can f 1 shows partial IgE cross-reactivity with its human homologue tear lipocalin (hTL)^11^ and with the cat allergen Fel d 7^12^. A cross-sectional and longitudinal study involving 779 randomly collected serum samples from children of the BAMSE birth cohort showed that sensitization to Can f 1 at the age of 4 years is a strong risk factor for the development of respiratory allergy at the age of 16 years^13^. In that study rCan f 1 showed a higher positive predictive value for the development of dog allergy than dog allergen extract.

While the T cell response of allergic patients to Can f 1 has been studied in great detail using overlapping peptides^14,15^ almost no information is available regarding the binding of allergic patients’ IgE to Can f 1. However, the detailed knowledge of IgE- and T cell epitopes is important for the development of new forms of allergen-specific immunotherapy (AIT)^16^. AIT is the only allergen-specific form of treatment with disease-modifying and long-lasting effects^17,18^. Furthermore there is evidence that it is clinically more effective than pharmacotherapy and also cost effective^19^. However, AIT for dog allergy is less efficient than AIT for cat allergy^20^ possibly due to the poor quality of the dog allergen extracts^21^. Recently a new strategy for AIT based on carrier-bound B cell epitope containing allergen peptides has been described^22^. As exemplified for a recombinant grass pollen allergy vaccine, non-allergenic peptides derived from the IgE binding sites of the major four grass pollen allergens have been grafted onto the PreS protein from hepatitis B as carrier^23^. This vaccine, BM32, has been shown to have no allergenic activity *in vitro* and *in vivo* by skin testing of allergic patients^24^. Recently the clinical efficacy of BM32 has been shown in an AIT study performed in a grass pollen exposure chamber^25,26^. The engineering of new B cell epitope- as well as of T cell-epitope based allergy vaccines requires the knowledge of IgE and T cell epitopes of major allergens^16^. We therefore investigated the IgE binding sites of the major dog allergen Can f 1. Using six overlapping peptides of 30 to 36 amino acids that cover the Can f 1 sequence peptide-specific antibodies were raised in rabbits. Each peptide was synthesized in two forms with cysteine residues for coupling to Keyhole Limpet Hemocyanin (KLH) either at their N- or C-terminus to analyze if the coupling orientation may influence the immunogenicity of the peptides. With the synthetic peptides and dog allergic patients’ sera the presence of sequential/linear IgE epitopes in Can f 1 was investigated. Furthermore, peptide-specific antibodies were used for competing with the allergic patients’ IgE binding to Can f 1 to search for conformational/discontinuous IgE epitopes. These two approaches combined with a molecular modelling approach enabled us for the first time to identify the major IgE binding site as a conformational epitope on the surface of a 3-dimensional model of Can f 1.

## Results

### Characterization of rCan f 1 and Can f 1-derived peptides

**Figure 1:**
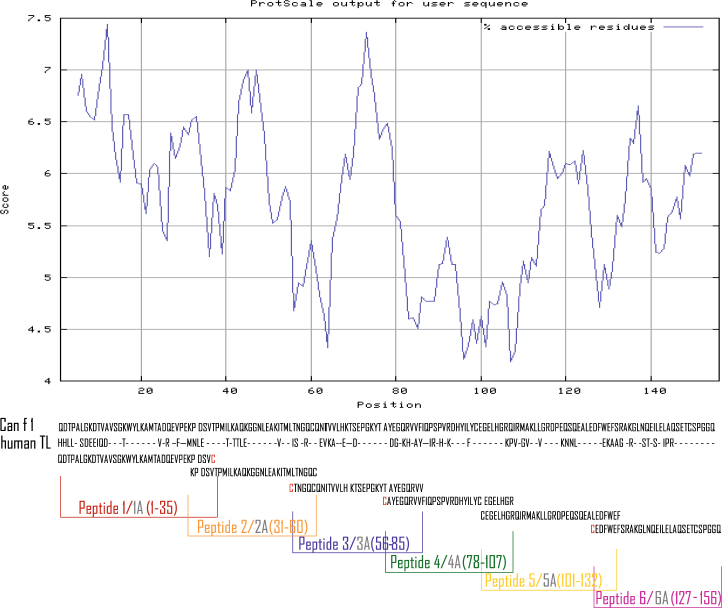
Surface accessibility plot of Can f 1. Regions (x-axis) with high surface accessibility show scores greater than 5.5 (y-axis). Middle: Sequence alignment of Can f 1 and human tear lipocalin (human TL). Dashes represent identical amino acids. Synthetic Can f 1–derived peptides are indicated at the bottom. Cysteins added for coupling purposes are marked in red. Each peptide was synthesized in two forms with Cystein at either C or N terminus (P1 corresponds to P1A, P2 to P2A, P3 to P3A, P4 to P4A, P5 to P5A, P6 to P6A). The sequences of the peptides can be found in Supplementary Table 1.

As a reference molecule rCan f 1 was expressed in *E.coli* and purified by affinity chromatography to more than 90% purity (see Supplementary Fig. S1 in the Repository). The circular dichroism (CD) analysis showed a folded protein predominantly consisting of β-sheets (see Supplementary Fig. S1). The calculated mass for rCan f 1 corresponded to the mass determined by matrix-assisted laser desorption/ionization time-of-flight analysis (MALDI-TOF) (18553.73 Da) (data not shown). The sequence of rCan f 1 that was expressed is identical to the sequence that was deposited in the International Union of Immunological Societies (IUIS) database (http://www.allergen.org/) except for one single amino acid difference (W instead of R at position 130 in the Can f 1 sequence) (Fig. 1).

Solvent accessibility prediction indicated that the regions of Can f 1 with predicted high surface accessibility can be covered with 5 partly overlapping peptides (P1, P2, P3, P5, and P6) (Fig. 1). Peptide P4 is derived from a region with low external accessibility with a part of its calculated solvent exposed surface stemming from hydrophobic residues lining the inside of the binding pocket but was included in the study to cover the whole Can f 1 sequence. Additional peptides (P1A, P2A, P3A, P4A, P5A and P6A) (Fig. 1, Supplementary Table S1) were synthesized with cysteine residues for coupling to KLH either at their N- or C-terminus to analyze if the coupling orientation may influence the immunogenicity of the peptides. The mass and identity of each of the synthetic peptides was confirmed by MALDI-TOF analysis and the peptides lacked any fold as shown by circular dichroism analysis (data not shown). Supplementary Table S1 in the Online Repository summarizes the position, length, and biochemical properties of the Can f 1-derived synthetic peptides. All the peptides were highly soluble in water and biological buffers with the exception of P4 and P4A which were derived from the hydrophobic region of Can f 1. P4 and P4A were soluble only in organic solvents but not in aqueous solutions. The coupling of peptides to KLH was achieved via cysteine residues whereas P4 and P4A could not be coupled due to their high hydrophobicity. The average relative surface exposures of the amino acids within the peptides were calculated according to the three-dimensional structure of human tear lipocalin (Supplementary Fig. S2). The following results were obtained for the regions covered by the peptides: Peptide 1A = 7: 51.4%, peptide 2A = 8: 61.8%, peptide 3A = 9: 63.2%, peptide 4A = 10: 59.2%, peptide 5A = 11: 61.2% and peptide 6A = 12: 70.6%. In addition the relative surfaces of the peptides (i.e., peptide surface/whole protein surface × 100) were calculated yielding the following results: Peptide 1: 18.5%, peptide 2: 19.5%, peptide 3: 21.7%, peptide 4: 19.8%, peptide 5: 24.5% and peptide 6: 23.4%.

### Can f 1-derived peptides lack IgE reactivity and allergenic activity

**Figure 2:**
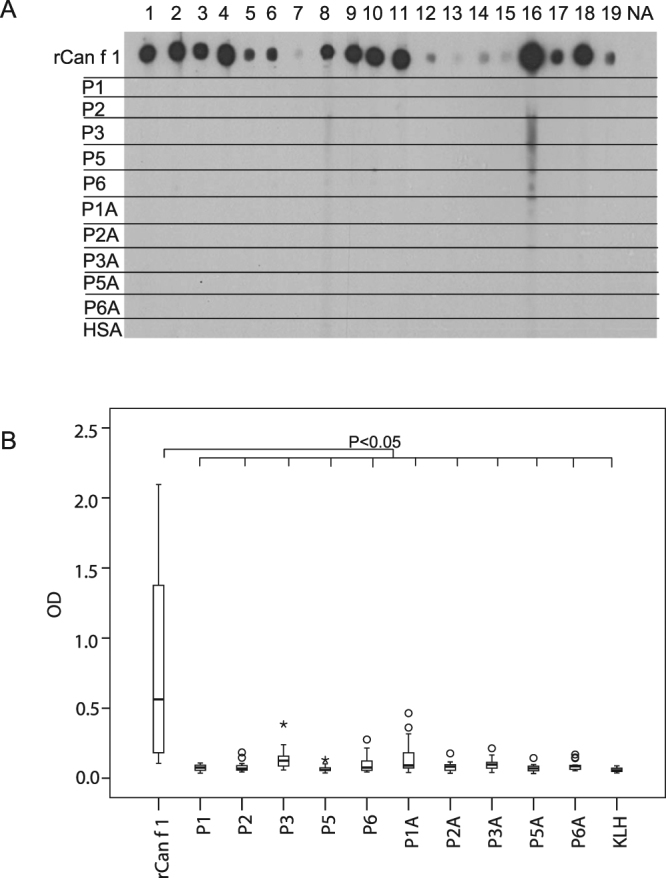
IgE reactivity of rCan f 1 and Can f 1-derived peptides. Dot-blotted rCan f 1, Can f 1-derived synthetic peptides (P1-P6, P1A-P6A) and HSA were tested for IgE reactivity with sera from 19 dog allergic patients (lanes 1–19) and a serum from a non-allergic individual (NA). Bound IgE antibodies were detected with ^125^I-labelled antihuman IgE abs and visualized by autoradiography **(A)**. Different exposures can be found in the Supplementary Fig. 3. IgE reactivity of dog-allergic patients to rCan f 1 and to KLH-coupled Can f 1-derived peptides was tested by ELISA in 19 patients **(B)**. OD values correspond to bound IgE and are represented in box plots, where boxes mark the interquartile range containing 50% of the data and lines across the boxes indicate the median. ○ represent outliers and * are extreme values.

**Figure 3:**
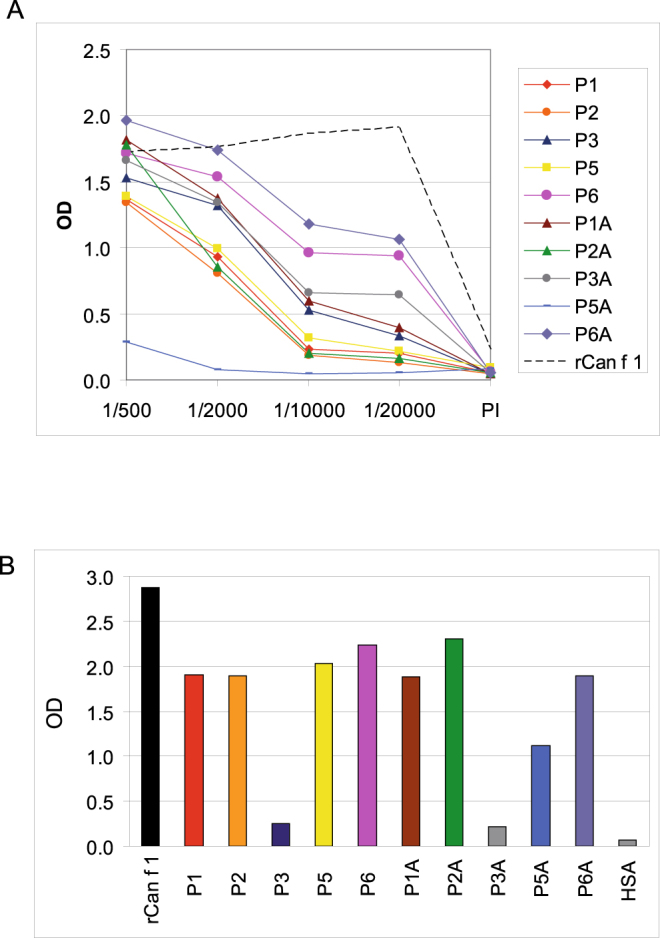
Titration of anti-peptide IgG antibodies specific for rCan f 1. Different dilutions (x-axis) of antisera obtained by immunization of rabbits with KLH-coupled Can f 1-derived peptides (P1-P6, P1A-P6A) and with rCan f 1 for comparison were reacted with ELISA plate-bound rCan f 1. OD values corresponding to bound rabbit antibodies are shown on the y-axis. PI: Pre-immune serum **(A)**. Reactivity of rabbit anti-Can f 1 antibodies (dilution 1:2000) with Can f 1-derived peptides (P1-P6, P1A-P6A), rCan f 1 and HSA. Shown are the OD values corresponding to bound IgG antibodies (y-axis) **(B)**. Results in A and B are means of duplicates with a deviation <5%.

In a first set of experiments, the IgE binding capacities of the 10 Can f 1-derived peptides were compared with that of rCan f 1 in a non-denaturing, dot blot assay. Figure 2A and Supplementary Fig. S3 show IgE reactivity obtained with sera from 19 dog allergic patients (1–19) and one non-allergic subject (NA). We found that each of the allergic patients’ sera showed IgE reactivity of varying intensity to rCan f 1, but none of the patients reacted with any of the Can f 1-derived peptides. The presence of the peptides and of rCan f 1 on the membrane was demonstrated with amido black staining (data not shown). Next we investigated if coupling of the peptides to KLH might render them IgE reactive. For this purpose KLH-coupled peptides were immobilized to ELISA plates and tested for IgE reactivity with sera from the same patients in comparison to rCan f 1 (Fig. 2B). We found that only few allergic patients’ sera showed weak IgE reactivity to KLH-coupled Can f 1-derived peptides. The residual IgE reactivity was significantly reduced for each peptide as compared to Can f 1 (p < 0.05, Fig. 2B). Coupling of the peptides to the ELISA plates was ensured by the demonstration that they react with Can f 1- and peptide-specific antibodies (Fig. 3, Table 1).

**Table 1:** Reactivity of the anti-Can f 1 antiserum and of antisera raised against Can f 1 peptides with rCan f 1 and Can f 1-derived peptides.

	coated											
	rCan f 1	P1	P2	P3	P5	P6	P1A	P2A	P3A	P5A	P6A	HSA
anti-Can f 1	**2.871**	**1.903**	**1.890**	**0.254**	**2.030**	**2.233**	**1.879**	**2.302**	**0.218**	**1.119**	**1.893**	0.069
anti-P1	**1.562**	0.078	0.109	0.083	0.080	0.074	**1.844**	**2.067**	**1.350**	**1.369**	0.082	0.068
anti-P2	**1.419**	**1.621**	**1.721**	0.070	0.078	0.070	0.073	0.074	0.130	0.090	0.089	0.064
anti-P3	**2.500**	0.077	0.076	**2.189**	0.104	0.090	0.073	0.067	**2.077**	0.078	0.081	0.062
anti-P5	**1.804**	0.070	0.076	0.212	**2.380**	**1.225**	0.113	0.071	0.065	**2.494**	**0.637**	0.064
anti-P6	**2.656**	0.068	0.084	0.071	**1.510**	**2.496**	0.083	0.074	0.067	**1.747**	**2.727**	0.071
anti-P1A	**2.172**	0.069	0.069	0.064	0.123	0.066	**2.518**	**2.365**	0.118	0.175	0.079	0.070
anti-P2A	**1.400**	0.093	0.070	0.070	0.086	0.075	**1.291**	**1.862**	0.088	0.093	0.073	0.068
anti-P3A	**1.826**	0.071	0.079	**1.937**	0.067	0.065	0.070	**1.290**	**2.318**	0.133	0.086	0.074
anti-P5A	**0.196**	0.077	0.078	0.082	**0.660**	**0.507**	0.070	0.065	0.065	**0.744**	**0.437**	0.065
anti-P6A	**2.384**	0.083	**2.299**	0.076	**1.512**	**2.446**	0.107	**1.316**	0.118	**1.580**	**2.489**	0.069

Mean OD values of duplicates (deviation <5%) corresponding to bound rabbit antibodies are shown. Positive results are indicated in bold.

In order to test whether the residual IgE reactivity of the KLH-coupled peptides may lead to allergenic activity of the conjugates we performed basophil activation studies. We found that Can f 1 induced release of β-hexosaminidase from basophils loaded with serum IgE from Can f 1-allergic patients whereas the equimolar mix of P6 and P1A and an equimolar mix of the KLH-coupled peptides did not activate the basophils (data not shown).

### Induction of Can f 1-specific IgG responses in rabbits with KLH-coupled Can f 1-derived peptides

In a first set of experiments we studied if directional coupling of Can f 1-derived peptides via cysteine residues placed either at the N- or C-terminal end of the peptides to KLH may affect the binding of antibodies induced by immunization with the peptide-KLH conjugates to Can f 1. For this purpose we synthesized each of the Can f 1-derived peptides with cysteine residue on the N-terminus and C-terminus (Fig. 1, Supplementary Table S1), coupled them to KLH and immunized rabbits with the conjugates. Figure 3A shows the Can f 1-specific IgG antibody levels of the peptide-specific antisera in titration experiments. The comparison of Can f 1-specific IgG levels induced by immunization with conjugates made via a C- versus N-terminal cysteine residue revealed differences regarding the induction of Can f 1-specific IgG antibodies for certain peptide conjugates. For example, we found that the peptide representing the N-terminal 35 amino acids of Can f 1 induced higher Can f 1-specific IgG responses when coupled via a N-terminal cysteine residue (i.e., peptide 1A) as compared to the same peptide coupled via a C-terminal cysteine residue (i.e., peptide 1) (Fig. 3A). A similar observation was made for the peptide covering amino acids 31–60 of Can f 1 (i.e., peptides 2 and 2A) and for the peptide including amino acids 101–132 of Can f 1 (i.e., peptides 5 and 5A) (Fig. 3A). Almost no Can f 1-specific antibodies were induced with peptide 5A when it was coupled via a C-terminal cysteine residue whereas peptide 5 coupled via a N-terminal cysteine induced a robust Can f 1-specific IgG response (Fig. 3). No such difference was noted for the peptide spanning amino acids 56–85 (i.e., peptides 3 and 3A). Regarding the peptide comprising the C-terminal end of Can f 1 (i.e., peptides 6 and 6A) the peptide coupled via the C-terminal end (i.e., peptide 6A) induced slightly higher Can f 1-specific IgG levels than the peptide coupled via the N-terminal cysteine (i.e., peptide 6) (Fig. 3A).

The analysis of the fine specificities of the antisera induced with the coupled peptides showed that the peptide antisera reacted with the isolated peptides against which they were raised regardless if the cysteine residue for coupling was added at the N- or C-terminus and for certain antisera reactivity with peptides containing overlapping sequences were found (Table 1). Regarding the anti-peptide 1 antiserum we noticed that it reacted well with Can f 1 and peptide 1A but only weakly with peptide 1 which was used for immunization. The anti-P6A antiserum showed reactivity with peptides 2 and peptide 2A which might be attributed to the presence of a stretch of similar amino acids (**IL**EL**AQ…GQC** in peptide 6A and **IL**K**AQ**K…**GQC** in peptide 2A) in these peptides (Table 1, Supplementary Table S1). No reactivity of any of the tested antisera was observed with the control protein human serum albumin (Table 1).

### Antisera specific for Can f 1-derived peptides can be used to map IgE-binding sites on rCan f 1

**Figure 4:**
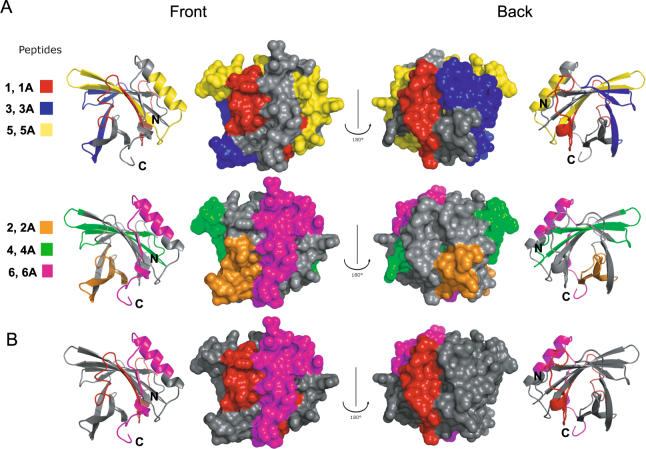
Mapping of Can f 1-derived peptides onto a model of the 3-dimensional structure of Can f 1 created according to the structure of human tear lipocalin (PDB: 1XK1). Views from two different sides (front and back side) are shown. Peptides were marked in different colors and were displayed in a ribbon (very left and very right panel) and surface representation (two middle panels) of Can f 1 **(A)**. Peptides 1/1A and 6/6A defining a major IgE-reactive patch are shown in red and purple, respectively **(B)**.

It has been shown for several important respiratory allergens that allergic patients IgE antibodies are directed against conformational epitopes on the folded allergen whereas allergen-derived peptides without fold lack IgE reactivity^27^. Nevertheless such peptides can be used to raise allergen-specific antibodies which can interfere with IgE binding to the folded allergen and allow mapping of the binding sites of allergic patients IgE antibodies using inhibition experiments^28^. Accordingly we tested the rabbit anti-peptide antibodies for their ability to inhibit the binding of allergic patients’ IgE to rCan f 1. Table 2 shows the degree of inhibition of patients’ IgE binding to rCan f 1 obtained by pre-incubation of the allergen with the individual peptide-specific antibodies and, for comparison, by pre-incubation with antibodies raised against the complete folded rCan f 1 for patients with dog allergy. The strongest inhibition of patients’ IgE binding to rCan f 1 was obtained with anti-P6, anti-P6A which were raised against the C-terminal Can f 1 peptide aa 127–156 and with anti-P1A antibodies directed against the N-terminal peptide aa 1–35 (Table 2). The mean inhibitions of IgE binding obtained with anti-P6, anti-P6A and anti-P1A were 45%, 40% and 39% respectively, while mean inhibition with antibodies raised against the complete Can f 1 allergen was 80%. The inhibitions obtained with the other anti-peptide antibodies were lower (P1: 19%; P2: 27%; P3: 28%; P5: 24%; P2A: 28%; P3A: 21%; P5A: 9%). These results indicate that IgE binding epitopes were predominantly present on the N- and the C-terminal portions of Can f 1. To study the possible contribution of the Can f 1 regions defined by the latter peptides to IgE binding we tested different mixes of anti-peptide antibodies for their ability to inhibit allergic patients IgE binding to rCan f 1. Results in Table 3 demonstrated that the mix of anti-P6 and anti-P1A antibodies gave the best inhibition results for most of the patients (mean inhibition 73%) that came very close to the inhibition obtained with anti-Can f 1 antibodies (mean inhibition 90%). When other anti-peptide antibodies were added to the mix of anti-P6 and anti-P1A antibodies (e.g., P6 + 1A + 2A + 3 + 5) only a marginal increase of inhibition of IgE binding was obtained (mean inhibition 80%). Interestingly, peptides P6 and P1A which in the primary sequence represent the C- and N-terminus of Can f 1 (Fig. 1) become adjacent peptides within a structural model generated for Can f 1 based on the three-dimensional structure of human tear lipocalin (Fig. 4). Thus they seem to be part of conformational IgE epitopes.

**Table 2:** Inhibition of patients (1–18) IgE binding to rCan f 1 by antisera specific for Can f 1 peptides.

	1	2	3	4	6	8	9	10	11	16	17	18	% mean inhibition
anti-Can f 1	79	89	58	90	73	91	70	90	92	72	70	90	**80**
anti-P1	23	27	18	30	13	24	20	34	19	0	7	10	**19**
anti-P2	14	16	21	41	18	30	39	42	16	37	21	27	**27**
anti-P3	38	37	10	55	20	39	21	29	32	6	15	28	**28**
anti-P5	33	32	13	36	23	37	0	26	24	18	15	32	**24**
anti-P6	58	65	45	62	30	52	46	49	44	12	24	48	**45**
anti-P1A	52	53	26	66	31	59	47	57	36	0	16	24	**39**
anti-P2A	17	12	28	37	23	15	33	25	35	38	33	35	**28**
anti-P3A	32	29	13	38	19	34	14	13	25	22	3	12	**21**
anti-P5A	8	8	4	3	2	0	5	24	3	21	16	19	**9**
anti-P6A	51	56	34	65	23	55	45	50	36	11	15	40	**40**

**Table 3:** Inhibition of patients (1–18) IgE binding to rCan f 1 by combinations of antisera specific for Can f 1 peptides.

	1	2	3	4	6	8	9	10	11	17	18	% mean inhibition
anti-Can f 1	93	92	82	94	83	96	86	96	95	82	93	**90**
anti-P6	60	60	44	67	44	58	59	63	56	34	52	**54**
anti-P1A	43	46	28	55	30	38	52	44	42	28	37	**40**
anti-P6 + 1A	77	78	66	84	59	80	77	84	73	54	73	**73**
anti-P6 + 1A + 2A	72	74	60	81	54	79	76	79	78	46	70	**70**
anti-P6 + 1A + 2A + 3	80	85	72	87	66	84	81	85	80	61	77	**78**
anti-P6 + 1A + 2A + 3 + 5	82	88	74	87	69	86	80	84	85	64	78	**80**

### IgE inhibition experiments identify a major IgE-reactive patch formed by the N- and C-terminal peptides on the surface of Can f 1

To reveal the spatial location of each of the peptides on a structural model of Can f 1, the peptide sequences were highlighted in the 3D-structural model of Can f 1 (Fig. 4). In addition, the surface-exposed portions of the peptides within the Can f 1 structural model were calculated (Supplementary Fig. S2). Peptide 6 which induced IgG antibodies that inhibited patients IgE binding occupied the largest continuous surface of Can f 1 with most of its amino acids being exposed on one side of Can f 1 (Fig. 4A. front, purple). The N-terminal and C-terminal portions of peptide 1A derived from the Can f 1 N-terminus (aa 1–35) were exposed on different sides of the molecule in a way that its C-terminal portion became adjacent to the patch defined by peptide 6 whereas the N-terminal portion appeared on the backside (Fig. 4A,B, red). Peptides 6 and 1A, despite their distant location in the primary Can f 1 sequence (i.e., N- and C-terminus) constitute a conformational IgE epitope which actually shows high sequence identity between Can f 1 and the cross-reactive cat allergen, Fel d 7 (Supplementary Fig. S4)^12^. A large portion of the hydrophobic peptide 4 was buried in the Can f 1 molecule, only the very N- and C-terminal ends protruded in the model which was in agreement with the surface prediction showing a large central hydrophobic portion (Fig. 4A, Supplementary Fig. S2: green). Regarding peptide 3, mainly the N-terminal portion appeared on the surface of Can f 1 on the opposite site of the area defined by peptide 6 (Fig. 4A, back, blue). The N-terminus of peptide 2A appeared in the vicinity of the C-terminus of peptide 1A close to the patch defined by peptide 1A whereas the C-terminal end of peptide 2A appeared on the other site which also agrees with the surface prediction indicating that peptide 2A contains a central hydrophobic portion which traverses the Can f 1 molecule (Fig. 4A,B, orange). Also peptide 5 appeared on two sides of Can f 1 due to a central hydrophobic part with its N-terminus close to the C-terminal portion of peptide 1A and its C-terminus close to peptide 6 (Fig. 4A, yellow Supplementary Fig. S2). Thus it seems that peptides 5 and 2A contribute to the IgE-reactive patch defined by peptide 6 and the C-terminal portion of peptide 1 A.

## Discussion

Can f 1 is not only a major dog allergen but was also found to be the key allergen associated with the risk of developing of allergic respiratory symptoms in adolescence in a large birth cohort study^13^. Novel AIT strategies based on recombinant allergen derivatives and synthetic peptides can precisely target the T cell and B cell response in allergic patients and have shown promising results in clinical trials^16^. While the T cell epitopes of Can f 1 have been extensively studied^14,15,29^ no data have been available regarding the IgE epitopes of Can f 1. In this study we investigated IgE epitopes of Can f 1 by two approaches. In the first approach we compared IgE reactivity of Can f 1 which we produced as a pure recombinant folded protein with synthetic peptides spanning the Can f 1 sequence. The Can f 1 peptides had a length of approximately 30 amino acids but lacked any fold or conformation. We found that dog allergic patients showed IgE reactivity only to complete and folded Can f 1 but not to any of the peptides, neither as isolated peptides nor when coupled to KLH. This result indicated that Can f 1, like many other important respiratory allergens does not contain relevant sequential/continuous IgE epitopes but rather contains conformational IgE epitopes. For the mapping of conformational IgE epitopes we use a technology which is based on an indirect epitope mapping strategy. Synthetic antigen-derived peptides are coupled to a carrier molecule and used to immunize rabbits to produce antibodies which recognize the peptide in the context of the complete protein^30–32^. When the peptide-derived amino acids on the protein surface are occupied by the peptide-specific antibodies a reduction of allergic patients’ IgE binding can be measured in an IgE inhibition experiment. Using this indirect IgE epitope mapping strategy we found that antibodies raised against the N-terminal peptide 1A and against the C-terminal peptide 6 inhibited consistently allergic patients IgE binding. When both anti-peptide antibodies were used for inhibition of IgE binding we achieved an inhibition of binding which was almost as strong (>70%) as that achieved with an antiserum raised against the complete folded Can f 1 protein indicating that the area defined by peptides 1A and 6 contains the majority of Can f 1 IgE epitopes. We then located the region defined by peptides 1A and 6 on a three-dimensional model of the Can f 1 structure built on the basis of the homologous human tear lipocalin. Surprisingly, we found that peptides 1 A and 6 which in the primary sequence of Can f 1 comprise the N- and C-terminus of the molecule appear in close proximity on the surface of Can f 1 suggesting that during the folding of the protein in solution the peptides fold together and form a conformational IgE epitope consisting of the two ends of the protein. A similar finding has been made earlier for birch pollen profilin, a highly cross-reactive allergen from birch in which also the N- and C-terminus of the protein form a conformational IgE epitope^33^. Also many other important respiratory allergens contain mainly such conformational IgE epitopes^34,35^. It is known that mast cell and basophil activation by allergens requires engagement of at least two IgE antibodies. We have shown that patients IgE binding to the major IgE epitope of Can f 1 can be partially inhibited by IgG antibodies directed either against peptide 6 (54% inhibition) or against peptide 1A (40% inhibition). When IgG antibodies against these two peptides are used in combination the inhibition of patients’ IgE binding increased to 73%. This additive effect indicates that IgE antibodies with at least two different specificities can bind to the region defined by peptides 6 and 1A in close vicinity. We have previously shown for the birch allergen Bet v 1 that indeed several different Abs can bind simultaneously to a relatively small patch of the Bet v 1 structure^32^. Likewise, we have visualized simultaneous binding of four IgE antibodies to four small IgE-reactive peptides inserted into the N-terminal part of horse myoglobulin^36^. Therefore we think that the area identified on Can f 1 by peptides 6 and 1A is large enough to accommodate binding of at least two different IgE antibodies.

Our finding is clinically important for at least two reasons: First, peptides 1A and 6 show close sequence similarity with the cat allergen, Fel d 7 which shows partial cross-reactivity to Can f 1 and thus we can assume that similar epitopes are involved in IgE recognition of the two allergens^12^. There is no relevant sequence homology of peptides 1A and 6 with the corresponding regions in the other lipocalin allergens (i.e., *Felis domesticus*; Fel d 4, *Equus caballus*; Equ c 1, *Canis familiaris*; Can f 6, Can f 2, Can f 4 and *Bos domesticus*; Bos d 2) which actually show no relevant IgE cross-reactivity with Can f 1, except some cross-reactivity between Can f 1 and Can f 2. However, these cross-reactivity data are based on IgE inhibition experiments performed with few sera^11,37^ or only on correlations of IgE reactivities to both allergens^38^. The second major clinical implication of our work is that the non-allergenic Can f 1 peptides 1A and 6 can induce blocking IgG antibodies upon immunization and thus should be useful for the construction of a hypoallergenic B cell epitope-based vaccine for therapeutic and eventually prophylactic vaccination against dog allergy^22^.

## Materials and Methods

### Allergic patients’ sera, recombinant Can f 1

IgE reactivity testing and allergenic activity testing by basophil activation assay were conducted with blood samples of adult dog allergic patients from Austria (n = 19) after having obtained informed consent of the patients with the approval of the local ethics committee (Medical University of Vienna, Austria, EK: 565/2007; 1641/2014). All experiments were performed in accordance with relevant guidelines and regulations. Dog allergic patients reported exacerbation of at least one of the following clinical symptoms upon exposure to dogs: conjunctivitis, rhinitis, asthma and allergic reactions of the skin (Supplementary Table S2). Serological analysis showed the presence of IgE antibodies (>0.35 kU_A_/L) to dog dander extract (e5) as measured by ImmunoCAP (Phadia, Uppsala, Sweden) and IgE antibodies to Can f 1 as determined by ELISA and ImmunoCAP ISAC (Thermofisher, Uppsala, Sweden)^10^. Table S2 provides an overview of demographic, clinical and serological characteristics of the dog allergic patients. Sera from three non-allergic individuals were included as controls.

The cDNA coding for the mature Can f 1 without a N-terminal signal sequence and with a C-terminal hexahistidine tag was obtained by Reverse transcription-polymerase chain reaction (RT-PCR) amplification (primer Can f 1_fwd: 5′-gcgcatatgcaggataccccagccttgggaaagg-3′ and primer Can f 1_rev:

5′-cgctcgagaactcagtgatgatgatgatgatgctgtcctcctggagagcaggtttcgctc-3′) from a dog salivary gland cDNA library. The PCR product was cut with Nde I and Xho I, purified by agarose gel electrophoresis and cloned into pET 17b vector (Novagen, Darmstadt, Germany). Recombinant Can f 1 (rCan f 1) was expressed in *E. coli* strain BL21 (DE3) (Stratagene, La Jolla, CA) and purified via its His tag by Nickel affinity chromatography under denaturing conditions according to the manufacturers protocol (Qiagen, Hilden, Germany). Purified rCan f 1 was refolded by dialysis against distilled water. The purity of the recombinant protein was analysed by SDS-PAGE, and the molecular mass was determined by matrix-assisted laser desorption/ionization time of- flight mass spectrometry (Bruker, Billerica, MA, USA). To verify the proper fold of rCan f 1 a circular dichroism analysis was performed as described^39^.

### Synthesis, KLH- coupling and characterisation of Can f 1 peptides, modelling of the Can f 1 structure

Six overlapping peptides spanning the Can f 1 sequence with a length between 30 and 36 amino acids were identified based on the prediction of surface exposure of amino acids as determined by ProtScale bioinformatics tool from the ExPASY server^40^ (http://web.expasy.org/protscale/) (Fig. 1). Peptides contained cysteine residues for coupling to KLH either at their N- (P3, P4, P5, P6, P1A and P2A) or at their C- termini (P1, P2, P3A, P4A, P5A and P6A) (Supplementary Table S1). Peptides were synthesized using an Applied Biosystems peptide synthesizer Model 433A (Foster City, CA, USA)^28^, were subsequently purified by preparative High-performance liquid chromatography (HPLC) (Dionex, Thermofischer Scientific, Waltham, MA, USA) and their sizes and identities were confirmed by mass spectrometry (Bruker, Vienna, Austria). Can f 1-derived peptides were coupled to keyhole limpet hemocyanin (KLH) (Pierce, ThermoFisher Scientific, Waltham, MA, USA) and purified using a conjugation kit according to the manufacturer’s instructions (Pierce). The concentration of KLH-conjugated peptides was measured using the Micro BCA Assay Kit (Pierce).

The model of the Can f 1 structure was created by using the web portal of Phyre2^41^ using the PDB model of the human tear lipocalin 1XKI^42^ as template (61% sequence identity). Relative surface exposures were calculated with MSMS^43^ in the USCF Chimera package^44^ using Gly-X-Gly tripeptides^45^ as reference states and plotted as a bar graph against the amino-acid sequence with α-helix and β-sheet forming residues marked as H or B respectively (Supplemental Fig. S2). Graphic depictions of the model were rendered with PyMOL (PyMOL Molecular Graphics System, Version 1.7.4; Schroedinger, NY).

### Testing IgE reactivity by means of non-denaturing, RAST-based dot blot and by ELISA

IgE reactivity of uncoupled Can f 1-derived peptides was determined by dot blot. For this purpose 0.5 µg aliquots of each peptide, of rCan f 1 and as a control human serum albumin (HSA) were dotted onto Whatman Protran nitrocellulose membrane (GE healthcare, Little Chalfont, UK). After blocking three times for 20 min with gold buffer (50 mM sodium phosphate [pH 7.4], 0.5% [v/v] Tween-20, 0.5% [w/v] BSA, and 0.05% [w/v] sodium azide), membranes were incubated with dog-allergic patients’ sera (1:10 in gold buffer) or with serum from a non-allergic person (1:10 in gold buffer) overnight at 4 °C. Bound IgE antibodies were detected with 1:15 diluted ^125^I-labeled anti-human IgE Abs (Demeditec Diagnostics, Kiel, Germany) and visualized by autoradiography (Kodak XOMAT film).

IgE reactivity to Can f 1 peptides after coupling to KLH was determined by direct ELISA. Five µg/ml of KLH coupled peptides, rCan f 1 and KLH, respectively, were coated onto ELISA plates (Nunc, Roskilde, Denmark) over night at 4 °C. After washing, plates were incubated with patients’ sera diluted 1:5 in TBST (50 mM Tris, 150 mM NaCl, 0.5% [v/v] Tween-20) and bound human IgE was detected with HRP-coupled goat anti-human IgE antibodies diluted 1:2500 (KPL, Gaithersburg, MD). The optical density (OD) values corresponding to bound antibodies were measured at 405 and 490 nm. All determinations were conducted as duplicates, and results were expressed as mean values. To determine the cut-off for IgE reactivity to rCan f 1, mean OD values obtained for sera from three non-allergic subjects on the same plate were multiplied by two and patients with OD values above were considered Can f 1 reactive.

### Rat basophil leukemia (RBL) assay for testing allergenic activity

To test the allergenic activity of the peptides, rat basophil leukemia cells (RBL) expressing human high-affinity IgE receptor FcεRI (1 × 10^5^/well) were loaded overnight with sera from the dog-allergic patients and, for control purposes, with the serum from one non-allergic individual at a dilution of 1: 10. Cells were washed three times with Tyrode’s buffer (Sigma, Vienna, Austria) and exposed to serial dilutions of allergen (0.001, 0.01 and 0.1 µg/mL of Can f 1, mix P6 + P1A and mix P6-KLH + P1A-KLH, respectively) for 1 h. Supernatants were analysed for β-hexosaminidase activity as described previously^46^. Experiments were carried out in triplicates, and results are presented as mean percentages of total β-hexosaminidase released after addition of 1%Triton X-100 +/− SE of the mean (SEM).

### Immunisation of rabbits, determination of Can f 1-specific IgG antibody titers and IgG epitope mapping

Peptide-specific IgG antibodies were obtained by immunizing rabbits three times (first booster injection after 4 weeks and a second booster injection after 7 weeks) with each of the KLH-conjugated peptides (200 µg/injection) and, for control purposes, with recombinant rCan f 1 (200 µg/injection) using once Freund’s complete and twice Freund’s incomplete adjuvant (Charles River, Chatillon sur Chalaronnne, France). Rabbit immune responses were analyzed by ELISA titrations. For the measurement of specific rabbit IgG antibodies, ELISA plates were coated overnight with 5 µg/mL rCan f 1. After blocking, the plates were incubated overnight with serial dilutions of the corresponding rabbit anti-sera, or serum from a non-immunized rabbit (1:500, 1:2.000, 1:10.000 and 1:20.000). Bound rabbit IgG antibodies were detected with a 1:1.000 diluted horseradish peroxidase–labelled donkey anti-rabbit IgG antiserum (Amersham Biosciences, Little Chalfont, UK).

IgG epitope mapping was performed by direct ELISA. Synthetic peptides (P1, P2, P3, P5, P6, P1A, P2A, P3A, P5A, P6A), rCan f 1 or HSA as controls were coated on the ELISA plates at a concentration of 3 µg/ml and after washing and blocking, anti-Can f 1 antiserum (1:2.000 dilution) was added. Bound rabbit IgG was detected with HRP-labelled donkey anti-rabbit IgG (Amersham Biosciences). The IgG epitope mapping ELISA experiments were repeated three times giving similar results. Results of the ELISA experiments are expressed as means of duplicate determinations with a deviation of <5%.

### IgE ELISA competition assay analyzing the inhibition of human IgE binding to rCan f 1 by peptide-specific rabbit IgG

ELISA plates (Nunc, Roskilde, Denmark) coated overnight with 1 µg/mL of rCan f 1 were pre-incubated for 24 hours with each of the anti-peptide antisera, anti-Can f 1 antiserum, or, for control purposes, with serum from a non-immunized rabbit (all in a dilution of 1:50) and then washed. After overnight incubation with sera from dog allergic patients (diluted 1:10), bound IgE antibodies were detected with horseradish peroxidase–labelled goat anti-human IgE antibodies (KPL, Gaithersburg, MD). The percentage reduction of IgE binding achieved by pre-incubation with rabbit antisera was calculated as follows: 100−(OD_I_/OD_P_) × 100). OD_I_ and OD_P_ represent optical density values after pre-incubation with the rabbit immune serum or normal rabbit serum, respectively.

### Statistical analysis and sequence comparison

For statistical analysis, the statistical program SPSS (2008; version 16.0 SPSS Inc, Chicago, USA) was used. P-values < 0.05 were considered significant. Differences between the groups were compared using Mann–Whitney U-test. For sequence alignment Clustal Omega -multiple sequence alignment program was used (http://www.ebi.ac.uk/Tools/msa/clustalo/). Sequences with homology to Can f 1 were found using BLAST.

### Data availability

The datasets generated during and/or analysed during the current study are available from the corresponding author on reasonable request.

## Electronic supplementary material

Supplementary Information

## References

[1] Konradsen JR (2015). Allergy to furry animals: New insights, diagnostic approaches, and challenges. J Allergy Clin Immunol..

[2] Nilsson OB, van Hage M, Grönlund H (2014). Mammalian-derived respiratory allergens - implications for diagnosis and therapy of individuals allergic to furry animals. Methods.

[3] Berge M, Munir AK, Dreborg S (1998). Concentrations of cat (Fel d1), dog (Can f1) and mite (Der f 1 and Der p1) allergens in the clothing and school environment of Swedish schoolchildren with and without pets at home. Pediatr Allergy Immunol.

[4] Arlian LG, Neal JS, Morgan MS, Rapp CM, Clobes AL (2001). Distribution and removal of cat, dog and mite allergens on smooth surfaces in homes with and without pets. Ann Allergy Asthma Immunol.

[5] Burbach GJ (2009). GA(2)LEN skin test study II: clinical relevance of inhalant allergen sensitizations in Europe. Allergy.

[6] Schmitz R, Ellert U, Kalcklösch M, Dahm S, Thamm M (2013). Patterns of sensitization to inhalant and food allergens - findings from the German Health Interview and Examination Survey for Children and Adolescents. Int Arch Allergy Immunol.

[7] Asarnoj A (2008). Sensitization to inhalant allergens between 4 and 8 years of age is a dynamic process: results from the BAMSE birth cohort. Clin Exp Allergy.

[8] Konieczny A (1997). The major dog allergens, Can f 1 and Can f 2, are salivary lipocalin proteins: cloning and immunological characterization of the recombinant forms. Immunology.

[9] Mattsson L, Lundgren T, Everberg H, Larsson H, Lidholm J (2009). Prostatic kallikrein: a new major dog allergen. J Allergy Clin Immunol.

[10] Curin M (2014). Microarrayed dog, cat, and horse allergens show weak correlation between allergen-specific IgE and IgG responses. J Allergy Clin Immunol.

[11] Saarelainen S (2008). Animal-derived lipocalin allergens exhibit immunoglobulin E cross-reactivity. Clin Exp Allergy.

[12] Apostolovic D (2016). The cat lipocalin Fel d 7 and its cross-reactivity with the dog lipocalin Can f 1. Allergy.

[13] Asarnoj A (2016). Sensitization to cat and dog allergen molecules in childhood and prediction of symptoms of cat and dog allergy in adolescence: A BAMSE/MeDALL study. J Allergy Clin Immunol.

[14] Immonen A (2005). T cell epitope-containing peptides of the major dog allergen Can f 1 as candidates for allergen immunotherapy. J Immunol.

[15] Immonen AK (2007). Use of multiple peptides containing T cell epitopes is a feasible approach for peptide-based immunotherapy in Can f 1 allergy. Immunology.

[16] Bush RK (2016). Advances in allergen immunotherapy in 2015. J Allergy Clin Immunol.

[17] Durham SR (1999). Long-term clinical efficacy of grass-pollen immunotherapy. N Engl J Med.

[18] Larché M, Akdis CA, Valenta R (2006). Immunological mechanisms of allergen-specific immunotherapy. Nat Rev Immunol.

[19] Cox L (2015). Allergy immunotherapy in reducing healthcare cost. Curr Opin Otolaryngol Head Neck Surg.

[20] Hedlin G (1991). Immunotherapy with cat- and dog-dander extracts. V. Effects of 3 years of treatment. J Allergy Clin Immunol.

[21] Curin M (2011). Skin prick test extracts for dog allergy diagnosis show considerable variations regarding the content of major and minor dog allergens. Int Arch Allergy Immunol.

[22] Valenta R, Campana R, Focke-Tejkl M, Niederberger V (2016). Vaccine development for allergen-specific immunotherapy based on recombinant allergens and synthetic allergen peptides: Lessons from the past and novel mechanisms of action for the future. J Allergy Clin Immunol.

[23] Focke-Tejkl M (2015). Development and characterization of a recombinant, hypoallergenic, peptide-based vaccine for grass pollen allergy. J Allergy Clin Immunol.

[24] Niederberger V (2015). Skin test evaluation of a novel peptide carrier-based vaccine, BM32, in grass pollen-allergic patients. J Allergy Clin Immunol.

[25] Zieglmayer P (2016). Mechanisms, safety and efficacy of a B cell epitope-based vaccine for immunotherapy of grass pollen allergy. EBioMedicine.

[26] Cornelius C (2016). Immunotherapy With the PreS-based Grass Pollen Allergy Vaccine BM32 Induces Antibody Responses Protecting Against Hepatitis B Infection. EBioMedicine.

[27] Valenta R (2010). From allergen genes to allergy vaccines. Annu Rev Immunol.

[28] Focke-Tejkl M (2014). Dissection of the IgE and T-cell recognition of the major group 5 grass pollen allergen Phl p 5. J Allergy Clin Immunol.

[29] Liukko AL (2014). Human CD4+ T cell responses to the dog major allergen Can f 1 and its human homologue tear lipocalin resemble each other. PLoS One.

[30] Arnon R, Van Regenmortel MH (1992). Structural basis of antigenic specificity and design of new vaccines. FASEB J.

[31] Focke M (2004). Non-anaphylactic surface-exposed peptides of the major birch pollen allergen, Bet v 1, for preventive vaccination. Clin Exp Allergy.

[32] Gieras A (2011). Mapping of conformational IgE epitopes with peptide-specific monoclonal antibodies reveals simultaneous binding of different IgE antibodies to a surface patch on the major birch pollen allergen, Bet v 1. J Immunol.

[33] Fedorov AA, Ball T, Mahoney NM, Valenta R, Almo SC (1997). The molecular basis for allergen cross-reactivity: crystal structure and IgE-epitope mapping of birch pollen profilin. Structure.

[34] Valenta R, Campana R, Marth K, van Hage M (2012). Allergen-specific immunotherapy: from therapeutic vaccines to prophylactic approaches. J Intern Med.

[35] Focke M, Swoboda I, Marth K, Valenta R (2010). Developments in allergen-specific immunotherapy: from allergen extracts to allergy vaccines bypassing allergen-specific immunoglobulin E and T cell reactivity. Clin Exp Allergy.

[36] Gieras A (2016). IgE epitope proximity determines immune complex shape and effector cell activation capacity. J Allergy Clin Immunol.

[37] Kamata Y (2007). Characterization of dog allergens Can f 1 and Can f 2. 2. A comparison of Can f 1 with Can f 2 regarding their biochemical and immunological properties. Int Arch Allergy Immunol.

[38] Perzanowski MS (2016). Relevance of specific IgE antibody titer to the prevalence, severity, and persistence of asthma among 19-year-olds in northern Sweden. J Allergy Clin Immunol.

[39] Curin M (2014). Hypoallergenic derivatives of Fel d 1 obtained by rational reassembly for allergy vaccination and tolerance induction. Clin Exp Allergy.

[40] Gasteiger, E. *et al*. Protein identification and analysis tools on the ExPASy server. In: Walker JM, editor. The proteomics protocols handbook. 1st ed. Totowa (NJ): Humana Press; p. 571–607 (2005).

[41] Kelley LA, Mezulis S, Yates CM, Wass MN, Sternberg MJE (2015). The Phyre2 web portal for protein modeling, prediction and analysis. Nat Protoc.

[42] Breustedt DA, Korndörfer IP, Redl B, Skerra A (2005). The 1.8-A crystal structure of human tear lipocalin reveals an extended branched cavity with capacity for multiple ligands. J Biol Chem.

[43] Sanner MF, Olson AJ, Spehner JC (1996). Reduced surface: an efficient way to compute molecular surfaces. Biopolymers.

[44] Pettersen EF (2004). UCSF Chimera - A visualization system for exploratory research and analysis. J Comput Chem.

[45] Bendell CJ (2014). Transient protein-protein interface prediction: datasets, features, algorithms, and the RAD-T predictor. BMC Bioinformatics.

[46] Hartl A, Hochreiter R, Stepanoska T, Ferreira F, Thalhamer J (2004). Characterizatio of the protective and therapeutic efficiency of a DNA vaccine encoding the major birch pollen allergen Bet v 1a. Allergy.

