# Supplementary material for: Clustering of conformational IgE epitopes on the major dog allergen Can f 1
Source: Sci Rep. 2017 Sep 22;7:12135. doi: 10.1038/s41598-017-11672-5 (PMC5610169; doi:10.1038/s41598-017-11672-5)
Supplement: Supplementary file 1 — Supplementary Information [file 41598_2017_11672_MOESM1_ESM.pdf]

Supplementary information

Clustering of conformational IgE epitopes on the major dog allergen Can f 1

*Mirela Curin[1], Milena Weber[1], Gerhard Hofer[2], Danijela Apostolovic[3], Walter Keller[2], Renate Reininger[4], Ines Swoboda[1], Susanne Spitzauer[4], Margit Focke-Tejkl[1], Marianne van Hage[3], Rudolf Valenta[1]**

[1]Division of Immunopathology, Department of Pathophysiology and Allergy Research, Center for Pathophysiology, Infectiology and Immunology, Medical University of Vienna, Vienna, Austria, [2]Institute of Molecular Biosciences, University of Graz, Graz, Austria, [3]Immunology and Allergy Unit, Department of Medicine Solna, Karolinska Institute and University Hospital, Stockholm, Sweden, [4]Department of Laboratory Medicine, Medical University of Vienna, Vienna, Austria

Supplementary Figures and Tables

Supplementary Figure S1. Characterization of rCan f 1. Coomassie-stained SDS–PAGE of purified rCan f 1. M, molecular weight marker (kDa) **(A)**. Fold analysis by circular dichroism. The mean residue molar ellipticities for rCan f 1 (y-axis: degree cm^2 $dmol^{-1}$) were recorded in the wavelengths range from 190 to 260 nm (x-axis) **(B)**.

Supplementary Figure S2. Surface exposed amino acids calculated for the Can f 1 peptides according to the three-dimensional structure of human tear lipocalin. Shown are the average relative surface exposures of amino acids of the Can f 1-derived peptides (x-axes) compared to the G-X-G tripeptide accessibility of each respective amino acid (y-axes). The Can f 1-derived peptides are indicated in colour.

Supplementary Figure S3. IgE reactivity of rCan f 1 and Can f 1-derived peptides. Dot-blotted rCan f 1 and HSA (lower panel, lanes 21-39) and Can f 1-derived synthetic peptides (P1-12) and HSA (upper panel, lanes 1-19) were tested for IgE reactivity with sera from 19 dog allergic patients and a serum from a non-allergic individual (lanes 20 and 40). Bound IgE antibodies were detected with ^{125}I-labelled antihuman IgE abs and visualized by autoradiography. Films with shorter and with longer exposure times are shown. P7 is named peptide 1A in the main text, P8 is P2A, P9 is P3A, P11 is P5A, P12 is P6A.

Supplementary Figure S4. Sequence comparison of Can f 1 with lipocalins from other species. Dashes indicate identical amino acids and the percentages of sequence identities are shown on the right side. Peptides 1A and 6 defining a major IgE-reactive patch of Can f 1 are boxed. Nomenclature of the allergens is according to WHO/IUIS Allergen Nomenclature Sub-Committee (www.allergen.org); *Felis domesticus, Equus caballus, Canis familiaris, Bos domesticus.*

Supplementary Table S1. Characterization of Can f 1-derived synthetic peptides.

Supplementary Table S2. Demographic, clinical and serological characteristics of dog allergic patients.

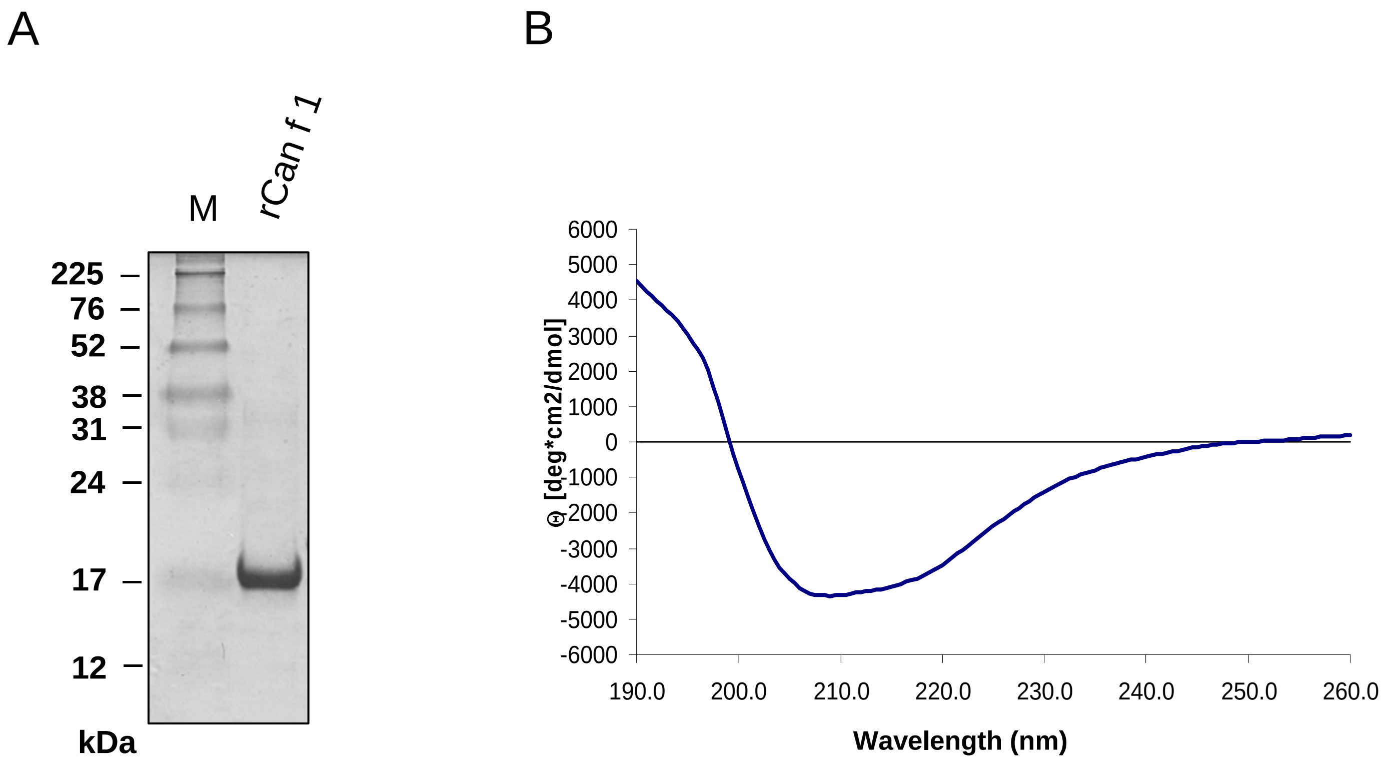

Supplementary Figure S1

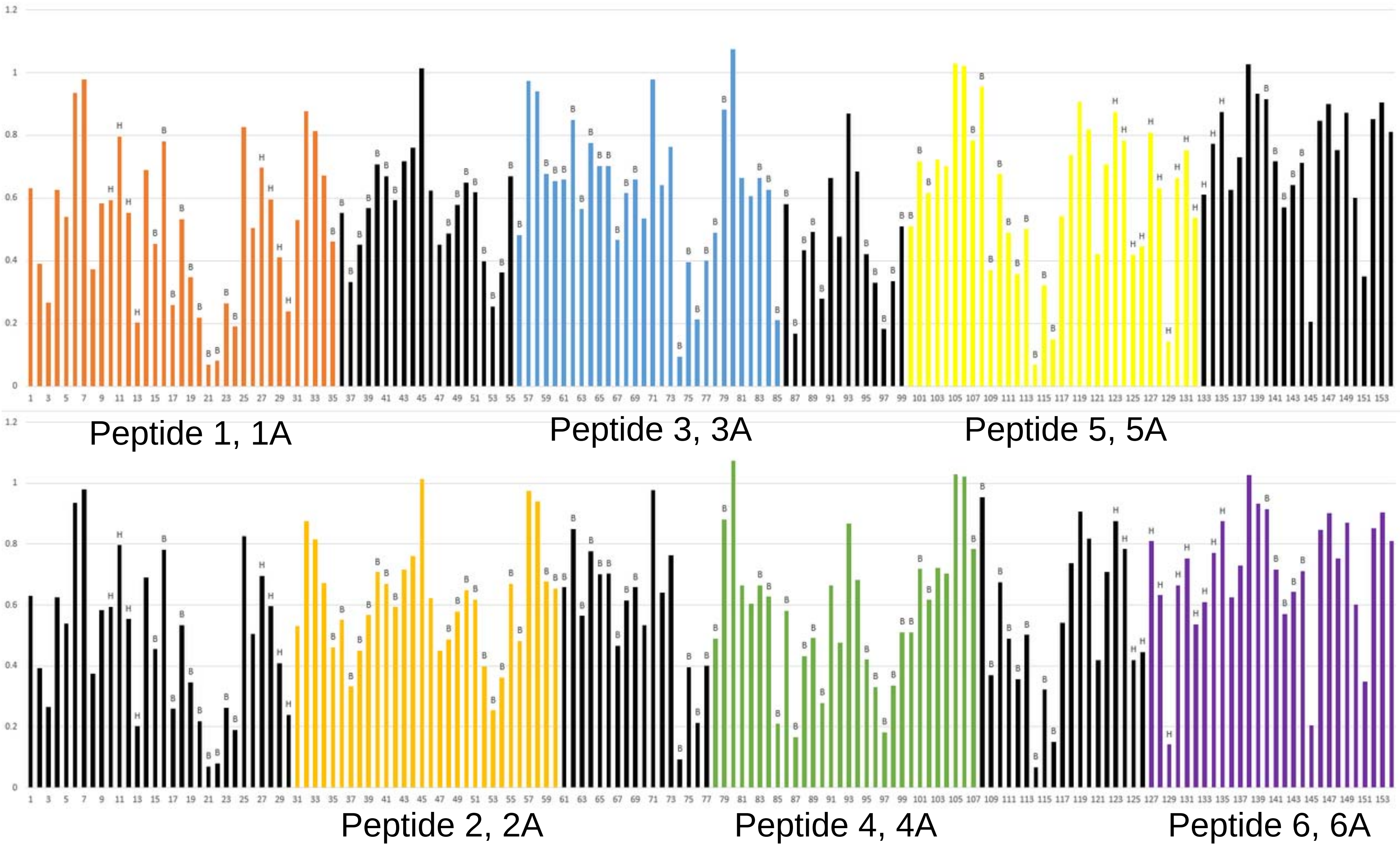

Supplementary Figure S2

shorter exposure time

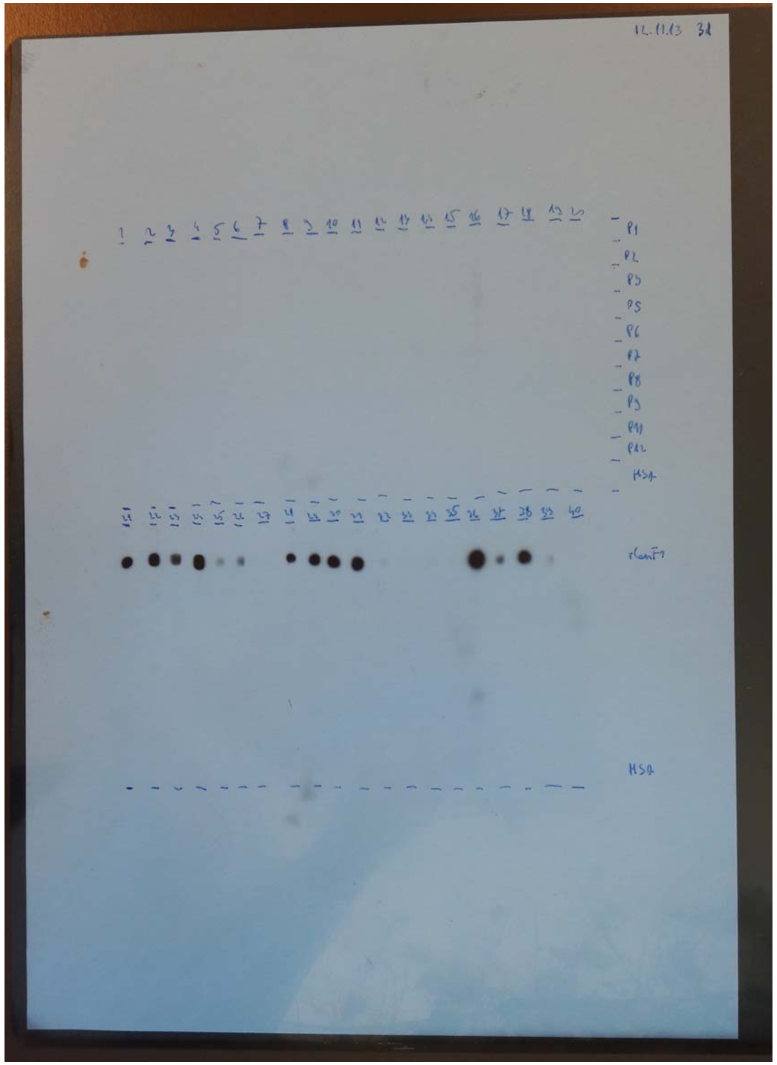

longer exposure time

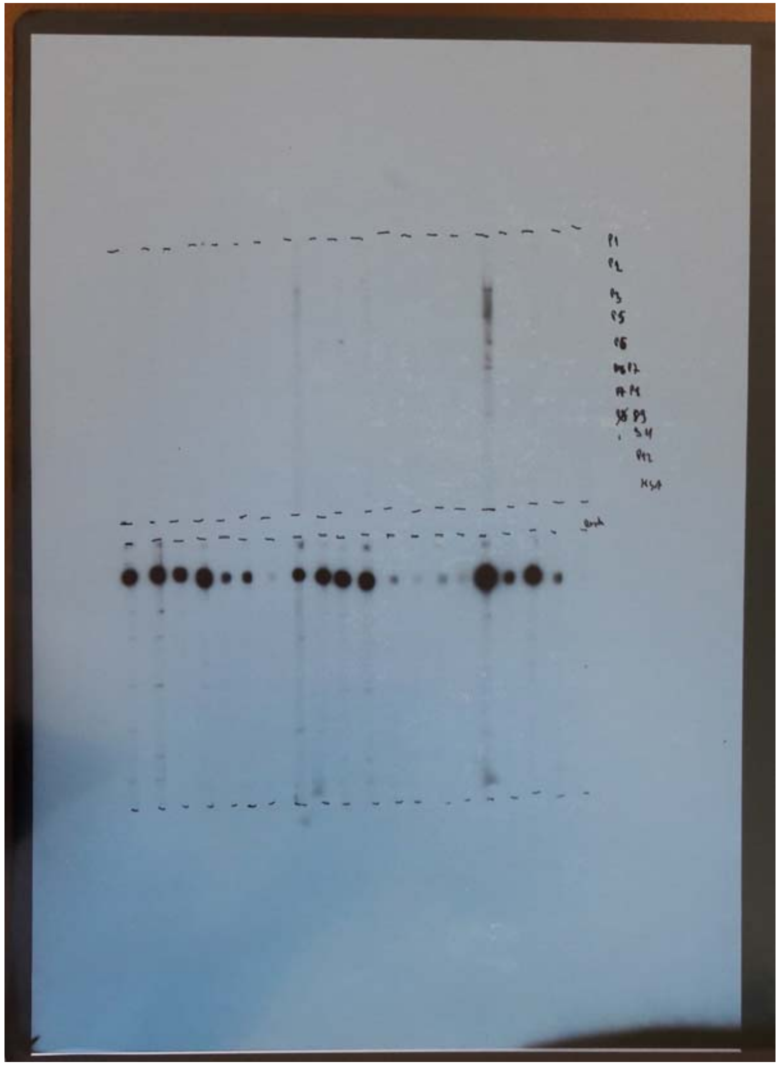

Supplementary Figure S3

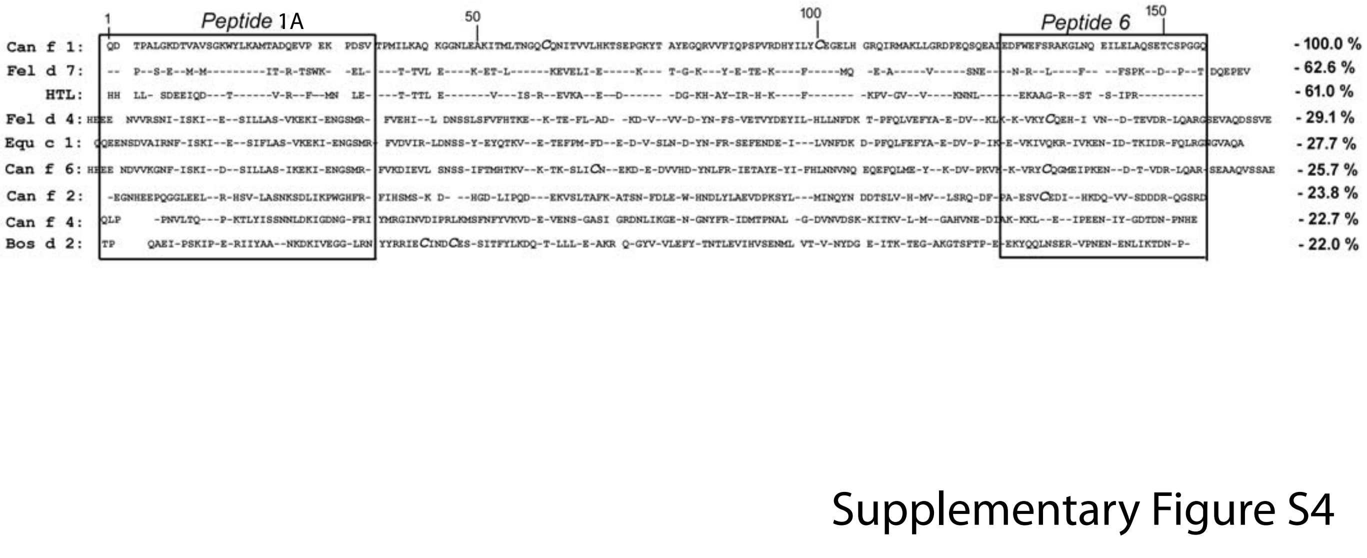

Supplementary Figure S4

Supplementary Table S1. Characteristics of synthetic Can f 1-derived peptides

Peptide	Position (aa)	Sequence	No. of amino acids	Molecular mass (Da)	Isoelectric point
peptide 1	1-35	QDTPALGKDTVAVSGKWYLKAMTADQEVPEKPDSV**C**	36	3879.37	4.53
peptide 2	31-60	KPDSVTPMILKAQKGGNLEAKITMLTNGQC	30	3187.78	9.11
peptide 3	56-85	**C**TNGQCQNITVVLHKTSEPGKYTAYEGQRVV	31	3424.85	8.03
peptide 4	78-107	**C**AYEGQRVVFIQPSPVRDHYILYCEGELHGR	31	3636.12	6.03
peptide 5	101-132	CEGELHGRQIRMAKLLGRDPEQSQEALEDFWEF	33	3919.36	4.59
peptide 6	127-156	**C**EDFWEFSRAKGLNQEILELAQSETCSPGGQ	31	3473.79	4.14
peptide 1A	1-35	**C**QDTPALGKDTVAVSGKWYLKAMTADQEVPEKPDSV	36	3879.37	4.53
peptide 2A	31-60	**C**KPDSVTPMILKAQKGGNLEAKITMLTNGQ	30	3187.78	9.11
peptide 3A	56-85	TNGQCQNITVVLHKTSEPGKYTAYEGQRVV**C**	31	3424.85	7.74
peptide 4A	78-107	AYEGQRVVFIQPSPVRDHYILYCEGELHGR**C**	31	3636.12	6.03
peptide 5A	101-132	EGELHGRQIRMAKLLGRDPEQSQEALEDFWEF**C**	33	3919.36	4.59
peptide 6A	127-156	EDFWEFSRAKGLNQEILELAQSETCSPGGQ**C**	31	3473.79	4.14

Cysteins added for coupling to KLH are marked in bold

Supplementary Table S2. Demographic, clinical and serological characteristics of dog allergic patients

Patient	Age	Sex	clinical symptoms upon contact with animals	Other allergies	Specific IgE level to e5 (kU_A/l)	Total IgE level (kU/L)
1	26	M	RH, CO, AS, AD	tp, fr, nu	12.7	>5000
2	31	F	AS, AD	tp, gp, hdm, fi, nu, cm	23.7	>2000
3	26	F	RH, CO, AS, U	tp, gp, hdm, fi, nu	14.6	570
4	47	F	RH, AS, AD	tp, gp, hdm, fi	>100	1224
5	23	M	RH, CO, AS	tp, gp, hdm, fr	16.4	316
6	26	M	CO, AD	tp, gp, hdm, he, nu	4.47	> 2000
7	20	M	AS	gp, hdm	9.97	1771
8	29	M	RH, CO, AS	tp, gp, hdm	63.0	>2000
9	30	F	RH, CO	nd	30.9	nd
10	75	F	RH, CO, AS, AD	tp, gp, hdm	>100	>2000
11	34	F	CO, AS, AD	tp, gp, hdm	78.1	>2000
12	37	M	RH, CO, AS, AD	tp, gp ,hdm	1.46	141
13	36	F	CO, AS	tp, gp, nu	nd	nd
14	35	M	RH, CO, AS	gp, hdm	nd	nd
15	36	M	RH, AS	hdm	nd	nd
16	42	F	RH, CO, AS	tp, gp, hdm, ve, he, cm	66.7	>2000
17	41	M	RH	tp, gp, ve	1.81	44
18	23	F	RH, CO, AS, AD	tp, gp, hdm, nu, ve, he, cm, mo	77.9	>2000
19	47	M	RH, CO, AS, U	tp, gp	20.1	420

Demographic data, clinical symptoms and sensitisation to other allergen sources are displayed for 19 dog-allergic patients. Total and allergen-specific IgE levels were measured by using ImmunoCAP and are displayed in kilo units/liter (kU/L) and kilo units of allergen/liter (kU_A/L), respectively. The cutoff value is 0.35 kU_A/L.

M, male; F, female; e5, dog dander extract; nd, not determined; RH, rhinitis; CO, conjuctivitis; AS, asthma; AD, atopic dermatitis; U, urticaria; tp, tree pollen; gp, grass pollen; hdm, house dust mite; nu, nuts; ve, vegetables, fr, fruits; he, hens egg; cm, cows milk; fi, fish; me, medicaments; mo, moulds.
